# Longitudinal profiling of human androgenotes through single-cell analysis unveils paternal gene expression dynamics in early embryo development

**DOI:** 10.1093/humrep/deae072

**Published:** 2024-04-15

**Authors:** X Vendrell, P de Castro, L Escrich, N Grau, R Gonzalez-Martin, A Quiñonero, M J Escribá, F Domínguez

**Affiliations:** Reproductive Genetics Department, Sistemas Genómicos-Synlab, Valencia, Spain; Research Department, IVIRMA Global Research Alliance, IVI Foundation—Reproductive Biology and Bioengineering in Human Reproduction, IIS La Fe Health Research, Valencia, Spain; Embryology Department, IVIRMA Valencia, Valencia, Spain; Embryology Department, IVIRMA Valencia, Valencia, Spain; Research Department, IVIRMA Global Research Alliance, IVI Foundation—Reproductive Biology and Bioengineering in Human Reproduction, IIS La Fe Health Research, Valencia, Spain; Research Department, IVIRMA Global Research Alliance, IVI Foundation—Reproductive Biology and Bioengineering in Human Reproduction, IIS La Fe Health Research, Valencia, Spain; Research Department, IVIRMA Global Research Alliance, IVI Foundation—Reproductive Biology and Bioengineering in Human Reproduction, IIS La Fe Health Research, Valencia, Spain; Embryology Department, IVIRMA Valencia, Valencia, Spain; Research Department, IVIRMA Global Research Alliance, IVI Foundation—Reproductive Biology and Bioengineering in Human Reproduction, IIS La Fe Health Research, Valencia, Spain

**Keywords:** uniparental, androgenote, androcyte, haploid human embryo, single-cell transcriptomic analysis, RNA-seq, early embryo development

## Abstract

**STUDY QUESTION:**

How do transcriptomics vary in haploid human androgenote embryos at single cell level in the first four cell cycles of embryo development?

**SUMMARY ANSWER:**

Gene expression peaks at the fourth cell cycle, however some androcytes exhibit unique transcriptional behaviors.

**WHAT IS KNOWN ALREADY:**

The developmental potential of an embryo is determined by the competence of the oocyte and the sperm. However, studies of the contribution of the paternal genome using pure haploid androgenotes are very scarce.

**STUDY DESIGN, SIZE, DURATION:**

This study was performed analyzing the single-cell transcriptomic sequencing of 38 androcytes obtained from 10 androgenote bioconstructs previously produced *in vitro* (de Castro *et al.*, 2023). These results were analyzed through different bioinformatics software such as g: Profiler, GSEA, Cytoscape, and Reactome.

**PARTICIPANTS/MATERIALS, SETTING, METHODS:**

Single cell sequencing was used to obtain the transcriptomic profiles of the different androcytes. The results obtained were compared between the different cycles studied using the DESeq2 program and functional enrichment pathways using g: Profiler, Cytoscape, and Reactome.

**MAIN RESULTS AND THE ROLE OF CHANCE:**

A wave of paternally driven transcriptomic activation was found during the third-cell cycle, with 1128 upregulated and 225 downregulated genes and the fourth-cell cycle, with 1373 upregulated and 286 downregulated genes, compared to first-cell cycle androcytes. Differentially expressed routes related to cell differentiation, DNA-binding transcription, RNA biosynthesis and RNA polymerase II transcription regulatory complex, and cell death were found in the third and fourth with respect to the first-cell cycle. Conversely, in the fourth cell cycle, 153 downregulated and 332 upregulated genes were found compared with third cell cycle, associated with differentially expressed processes related to E-box binding and zinc finger protein 652 (ZNF652) transcription factor. Further, significant overexpression of LEUTX, PRAMEF1, DUXA, RFPL4A, TRIM43, and ZNF675 found in androgenotes, compared to biparental embryos, highlights the paternal contributions to zygote genome activation.

**LARGE SCALE DATA:**

All raw sequencing data are available through the Gene Expression Omnibus (GEO) under accessions number: GSE216501.

**LIMITATIONS, REASONS FOR CAUTION:**

Extrapolation of biological events from uniparental constructs to biparental embryos should be done with caution. Maternal and paternal genomes do not act independently of each other in a natural condition. The absence of one genome may affect gene transcription of the other. In this sense, the haploid condition of the bioconstructs could mask the transcriptomic patterns of the single cells.

**WIDER IMPLICATIONS OF THE FINDINGS:**

The results obtained demonstrated the level of involvement of the human paternal haploid genome in the early stages of embryo development as well as its evolution at the transcriptomic level, laying the groundwork for the use of these bioconstructs as reliable models to dispel doubts about the genetic role played by the paternal genome in the early cycles of embryo development.

**STUDY FUNDING/COMPETING INTEREST(S):**

This study was funded by Instituto de Salud Carlos III (ISCIII) through the project ‘PI22/00924’, co-funded by European Regional Development Fund (ERDF); ‘A way to make Europe’. F.D. was supported by the Spanish Ministry of Economy and Competitiveness through the Miguel Servet program (CPII018/00002). M.J.E. was supported by Instituto de Salud Carlos III (PI19/00577 [M.J.E.]) and FI20/00086. P.dC. was supported by a predoctoral grant for training in research into health (PFIS PI19/00577) from the Instituto de Salud Carlos III. All authors declare having no conflict of interest with regard to this trial.

## Introduction

During the earliest stages of human embryo developmental programming, temporal regulation of gene expression requires an uneven contribution from maternal and paternal factors (McGrath and Solter, 1984; Leng *et al.*, 2019). Maternal mRNA transcripts that accumulate during oogenesis drive the initial species-specific gene expression patterns until zygote genome activation (ZGA). As the zygotic genome is transcribed, maternal transcripts are degraded (Yuan *et al.*, 2023), allowing the zygotic transcripts to facilitate embryonic genome activation (EGA) at the 4- to 8-cell stage, or later, in nonhuman species (Zhai *et al.*, 2022). The dynamic regulation of the human transcripts that drive the ZGA to EGA transition remain poorly understood as most of the knowledge is inferred from animal models. ‘Maternal effect genes’ are postulated to regulate the acquisition of developmental competence, epigenetic reprogramming, cell division, DNA repair, and EGA in humans (Vendrell, 2013; Innocenti *et al.*, 2022), however, the contribution of paternal genes to these processes is unclear.

The interplay of maternal and paternal genomes during the first mitotic divisions obscures the analysis of each progenitor’s contributions. Artificially generated uniparental human bioconstructs, also known as parthenogenotes (PG) and androgenotes (AG), have emerged as experimental models with identical genomes to the original oocyte or sperm, respectively, used to create them (Liao *et al.*, 2020). Despite the similar strategies used to generate these bioconstructs in animals and humans (Xu *et al.*, 2021), our previous experience with these uniparental models highlighted shorter cell division cycles in AG compared to PG and biparental human embryos (Escribá *et al.*, 2016). Specifically, human AG undergo a wave of novel transcription during the first cell cycle, reflecting the paternal contribution to the G2/M phase through genes related to cell division, or more specifically, lipid redistribution, histone structure, and tubulin synthesis. Alternatively, maternal transcripts lead the third and fourth cell cycles, activating genes associated with morphogenic progression, signal transduction cascades, intercellular communication, and post-transcriptional modifications (de Castro *et al.*, 2023).

This study aims to unveil the paternal contribution to early human embryo development by comparing the transcriptomic profiles and key biological processes of human AG with published normal biparental embryos from the first to the fourth cell cycle.

## Material and methods

### Ethical approval

This research was conducted at the Instituto Universitario IVI Valencia and IVI Foundation (Valencia, Spain). Human AG production and use was approved by the Institutional Review Board of the IVI Valencia (1604-VLC-031_ME). Written informed consent was obtained from participating gamete donors.

### Study design

This study was performed using the single-cell (androcyte) transcriptomic sequencing results from previously *in vitro* produced uniparental androgenote bioconstructs (de Castro *et al.*, 2023). In brief, androgenotes (AGs) were generated using metaphase II (MII) oocytes from healthy donors. These oocytes were subjected to enucleation, followed by intracytoplasmic sperm injection (ICSI), and then, cultured in time-lapse incubators. Once the AGs reached the desired developmental stage, they were disaggregated into single cells designated as androcytes. Due to the legislation that regulate experimentation with this type of samples in Spain, we were only allowed to carry out the culture of bioconstructs up to day 3 of development. Ten AGs were produced for meticulous evaluation by longitudinal single-cell sequencing, comprising bioconstructs at the one-cell stage (first cell cycle; 1.1, 1.2, 1.3, and 1.4), with four androcytes (3.1 and 3.3) and five androcytes (3.2) in the third cell cycle, and with eight androcytes in the fourth cell cycle (4.1, 4.2, and 4.3). Based on a total of 41 individual androcytes obtained, 38 were found to be viable and were subsequently subjected to RNA-seq analysis (Supplementary Fig. S1B).

### Oocyte origin and haploid AG production

AGs used to analyse the longitudinal transcription profile were created in our previous study (de Castro *et al.*, 2023). In brief, oocytes were collected from healthy donors, aged 18 to 35 years, by follicular puncture and aspiration with a standard ovarian stimulation protocol. After vitrification of 19 MII oocytes with the Cryotop method (Kuwayama, 2007), with minimal adjustments (Cobo *et al.*, 2019), they were thawed (Cobo *et al.*, 2016) and their survival was evaluated, showing a rate of 91.7% (Cobo *et al.*, 2019). Only MII oocytes with healthy ooplasm were selected for the production of single-parent androgen bioconstructs (AG). AGs were created using established protocols (Kono *et al.*, 1993, 1995) with slight modifications (Escribá *et al.*, 2016). Briefly, MII oocytes were enucleated by polarized light microscopy (Oosight^®^; Barcelona, Spain) (Grau *et al.*, 2011), followed by fertilization by ICSI with donor sperm. The resulting AG embryos, with a single pronucleus and without a second polar body in the perivitelline space (n = 14; 82% fertilization rate), were cultured for an additional three days, as a maximum.

### Embryo culture and androcyte isolation

AG embryos were cultured in either an EmbryoSlide^®^ (Vitrolife, Göteborg, Sweden) or a Geri^®^ plate (Merck, Darmstadt, Germany) using 25 µL a single-step Gems culture medium, overlaid with 1.2 mL of mineral oil (Cook, Barcelona, Spain) for three days. Imaging was performed at predefined intervals using a monochromatic Leica camera (with 1280 × 1024 pixels) at a magnification of 200X under constant culture conditions (37°C, 6% CO_2_, and 5% O_2_, using N_2_ as balance gas). The culture was terminated at the first, third, or fourth cell cycle, corresponding to the one-cell, four or five-cell, and eight-cell stages, respectively. Androcytes were isolated following established clinical protocols (Rubio *et al.*, 2007). This isolation process involved placing the constructs in a Ca^2+^ and Mg^2+^-free medium (G-PGD; Vitrolife, Göteborg, Sweden), perforating the zona pellucida using Octax NaviLaser technology (Herbron, Germany), and gently aspirating individual cells using a biopsy pipette with a 30 µm inner diameter (Humagen; CooperSurgical, Målov, Denmark).

### RNA extraction and library preparation

We collected 38 individual androcytes for RNA-seq analysis, representing various cell cycle stages: one-cell stage (n = 4), four or five-cell stages (n = 13), and eight-cell stage (n = 21). Samples were processed using the SMART-Seq V4 Ultra-Low Input RNA Sequencing kit (Clontech, CA, USA) with oligo-dT priming. Each sample was individually incubated with 2 μL of 10× Reaction Buffer and stored at −80°C until total RNA extraction. Following the manufacturer’s instructions, 10 pg of RNA was used to synthesize cDNA, using the 3′SMART-Seq CDS primer II (Clontech, CA, USA) and underwent 17 cycles of PCR amplification. The resulting cDNA was purified using AMPure XP magnetic beads (Illumina, San Diego, CA, USA), and cDNA integrity was assessed on a 2100 Bioanalyzer (Agilent Technologies, USA). The TruSight One Sequencing Panel Library (Illumina, San Diego, CA, USA) was used to fragment 50 ng of genomic DNA and construct RNA-seq libraries. A pool of RNA was created with 5 nM RNA per sample, and the library included barcodes and adapters for sequencing and sample identification.

### Single-cell RNA-seq and bioinformatic analysis

Libraries and samples were sequenced on Illumina HiSeq2500 and NovaSeq platforms with a 300-nt read length in a paired-end design (150 bp fragments). Quality assessment of the raw sequence data was conducted using FastQC. Reads were aligned and quantified using the Salmon algorithm with the GRCh38 reference genome. Correlation and differential expression analyses were performed using the DESeq2 program. On average, 45 276 912 reads were sequenced per sample, with approximately 48.77% successfully mapped. Raw counts were employed for the analysis of differentially expressed genes (DEGs). Functional enrichment analysis of biological pathways was conducted using g: Profiler (v. e104_eg51_p15_3922dba; Tartu, Estonia) through the g: GOSt tool for over-representation and gene set enrichment analyses. The analysis encompassed Gene Ontology (GO) pathways from KEGG, Reactome, and WikiPathways, miRNA targets from miRTarBase, regulatory motif matches from TRANSFAC, tissue specificity from Human Protein Atlas, protein complexes from CORUM, and human disease phenotypes from Human Phenotype Ontology. Only DEGs with a fold change (FC) ≥ |2| and a *P*-value < 0.05 were subjected to further analysis. An enrichment map of biological processes was generated using GSEA (v.4.1.0; San Diego, CA, USA) and visualized through Cytoscape (v.3.7.2; Bethesda, MD, USA). Lastly, Reactome (v.3.7; Tartu, Estonia) was employed to provide an overall perspective of the affected biological pathways and to compare them with previous research findings (Reimand *et al.*, 2019; Rivera-Egea *et al.*, 2021).

## Results

### Transcriptomic profiles of AG at the first versus third cell cycle

**Figure 1.**
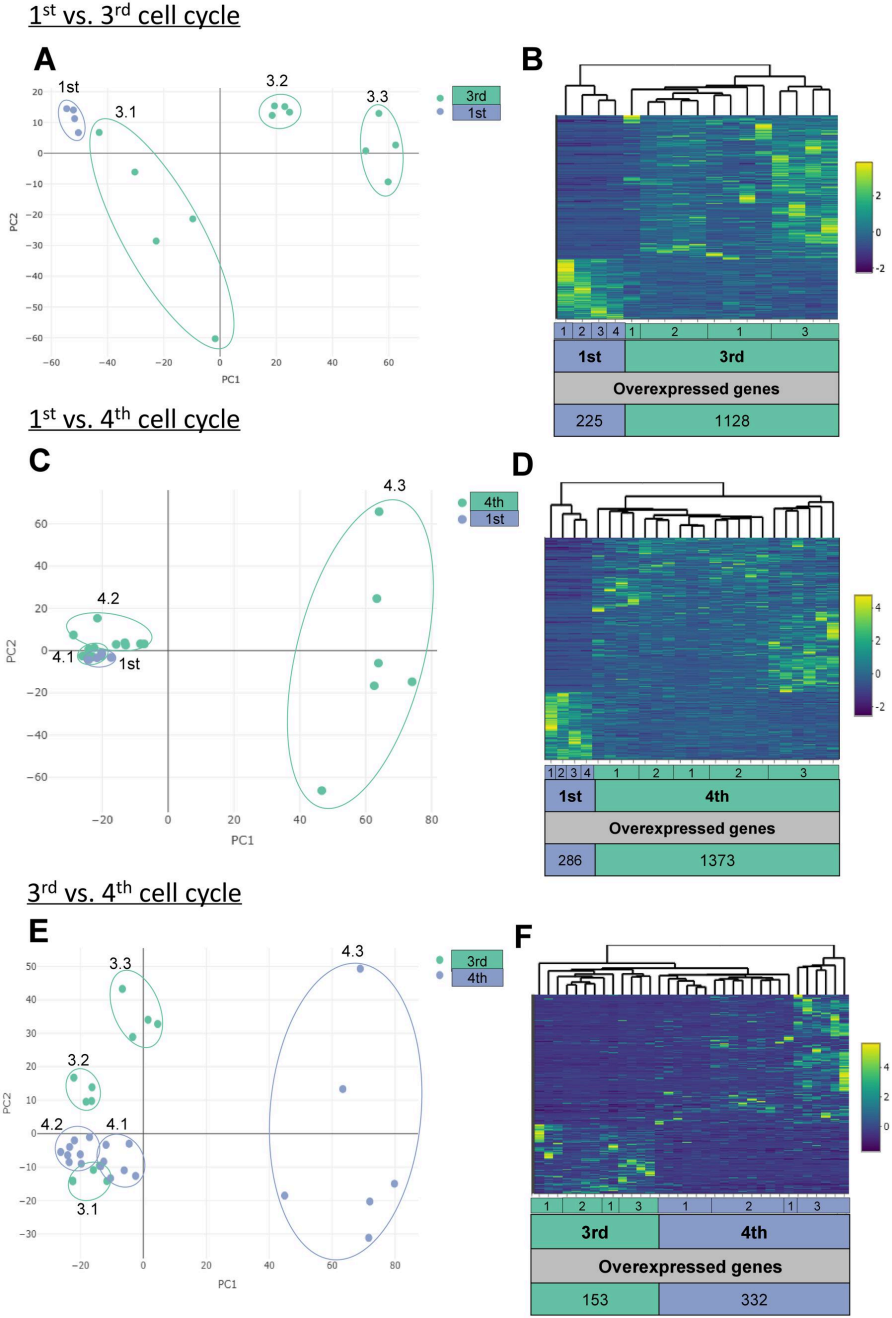
**Longitudinal transcriptomic profiling of androcytes.** Principal component analysis (PCA) plots where each dot represents the gene expression profile of individual androcytes isolated from androgenotes at the (**A**) first and third cell cycle (3.1, 3.2, and 3.3), (**C**) first and fourth cell cycle (4.1, 4.2, and 4.3), or (**E**) third and fourth cell cycle. (**B**, **D**, **F**) Corresponding heatmap and hierarchical clustering of the gene expression profiles depict the fold change (where upregulated genes are yellow and downregulated genes are blue) and highlight a large proportion of overexpressed genes. vs., versus.

**Figure 2.**
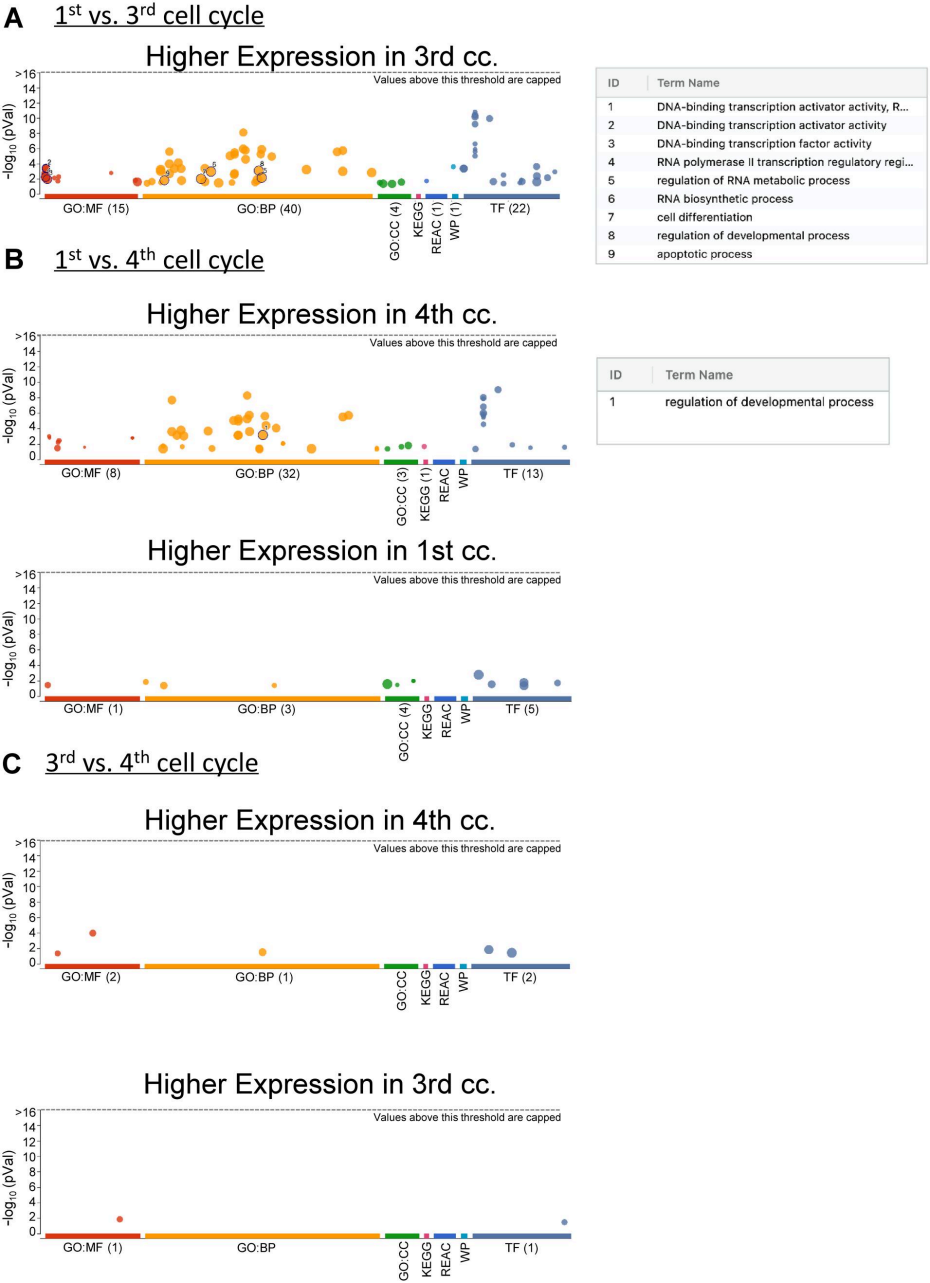
**Longitudinal functional enrichment analysis of androgenotes.** Manhattan plots showing the functional terms that were overexpressed in the (**A**) first versus third cell cycle (cc), (**B**) first versus fourth cc, (**C**) third versus fourth cc. The dot size is proportional to the number of significantly enriched genes in Gene Ontology (GO) molecular functions (GO: MF), biological processes (GO: BP), or cellular components (GO: CC); KEGG Pathways, Reactome (REAC), WikiPathways (WP) and Transfac (TF) databases (each represented by a different color). The numbered dots correspond with the ID assigned to the term names listed in the table on the right. vs., versus.

**Figure 3.**
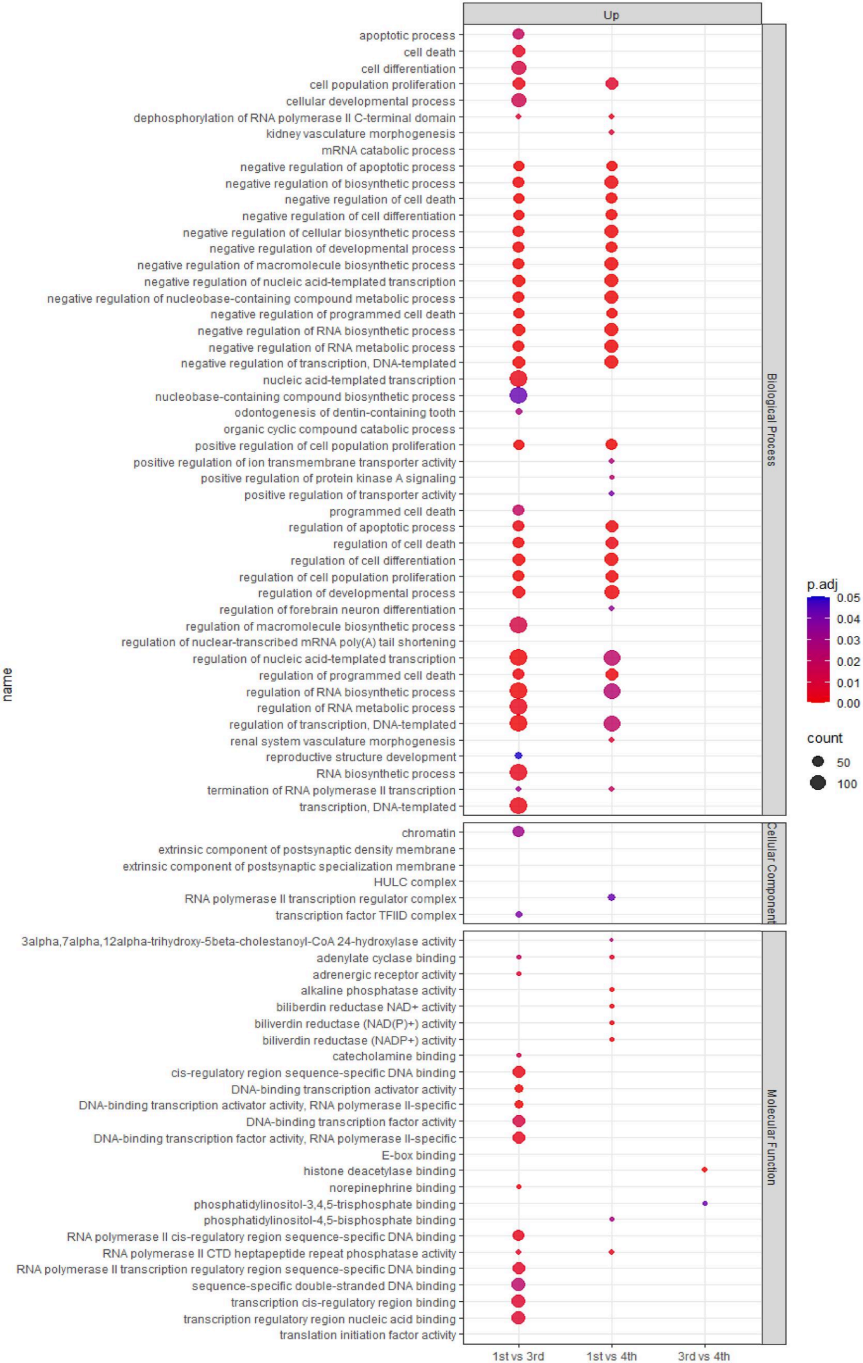
**Longitudinal Gene Ontology (GO) enrichment analysis of androgenotes.** Dot plot showing the differentially expressed GO biological processes, molecular functions and cellular component for each comparison (first versus third, first versus fourth, and third versus fourth cell cycle) on the *x* axis. The size of the dot represents the number of genes present in that category and the color of the dot represents the level of significance, with red and blue representing the highest and lowest significance, respectively.

Principal component analysis (PCA) and heatmap analysis revealed distinct gene expression patterns between the first and third cell cycle, with androcytes from the same AG grouped together (Fig. 1A and B). Notably, there were five times more significantly upregulated DEGs in the third cell cycle compared to the first (1128 versus 225 DEGs, respectively; Fig. 1B, Supplementary Table S1). Among the upregulated genes, 64% of them were significantly enriched (FC≥|2| and *P*-value < 0.05) in processes related to cell differentiation, promoting activity of DNA-binding transcription, RNA biosynthesis, among others (Figs 2A and 3; Supplementary Table S2). Similarly, the upregulated biological processes in the third cell cycle were mainly related to RNA, developmental and cell death processes (Supplementary Fig. S2). Meanwhile, no significant enrichment in GO categories and/or KEGG pathways were found related to the 225 significantly downregulated DEGs in the third cell cycle.

### Transcriptomic profiles of AG at the first versus fourth cell cycle

When comparing androcytes from the first and fourth cell cycle, PCA revealed distinct clustering patterns based on the AG stage (Fig. 1C). Interestingly, two of the three AGs from the fourth cell cycle (4.1 and 4.2) had more transcriptomic similarity with the first cell cycle than to their sibling AG (Fig. 1C). Nonetheless, both the heatmap and clustering analysis clearly distinguished the gene expression profiles of the two stages (Fig. 1D). Among the 1373 significantly upregulated DEGs at the fourth cell cycle (Fig. 1D, Supplementary Table S3), 68% were significantly enriched (FC≥|2| and *P*-value < 0.05) in processes mainly related to cell differentiation, the RNA polymerase II transcription regulatory complex, and cell death (Figs 2B and 3; Supplementary Table S4). Conversely, among the 286 significantly downregulated DEGs at the fourth cell cycle (Fig. 1D, Supplementary Table S3), 76% were significantly enriched (FC≥|2| and *P*-value < 0.05) in processes mainly related to mRNA catabolic processes, the HULC complex, translation initiation factor activity (Figs 2B and 3; Supplementary Table S4). Similar to the third cell cycle stage, these DEGs were significantly enriched in biological processes related to RNA, developmental and cell death processes (Supplementary Fig. S2).

### Transcriptomic profiles of AG at the third versus fourth cell cycle

With the exception of two three and four cell cycle androcytes overlapping, the PCA differentiated the androcytes according to their corresponding AG (Fig. 1E). While these nuances were reflected in the heatmap clustering analysis (Fig. 1F), it was still possible to distinguish the cell cycle stages, though not as clearly as in the previous comparisons. In this case, 332 DEGs were significantly upregulated between the fourth and third cell cycles (Supplementary Table S5). Among these, 75% were significantly enriched (FC≥|2| and *P*-value < 0.05) in processes related to histone deacetylases and phosphatidylinositol-3,4,5-trisphosphate binding, among others (Figs 2C and 3, Supplementary Table S6). Conversely, among the 153 DEGs that were significantly downregulated between the fourth and third cell cycle stage (Supplementary Table S5), 78% were significantly enriched (FC≥|2| and *P*-value < 0.05) in processes related to E-box binding and the zinc finger protein 652 (ZNF652) transcription factor (Figs 2C and 3, Supplementary Table S6).

### Transcriptomic comparison of key genes with respect to biparental embryos

An analysis of key genes belonging to the categories of pluripotency, zygote genome activation (ZGA) and embryo developmental progression in androgenotes was carried out and the results were compared with biparental embryos using previously published data (Yan *et al.*, 2013).

**Figure 4.**
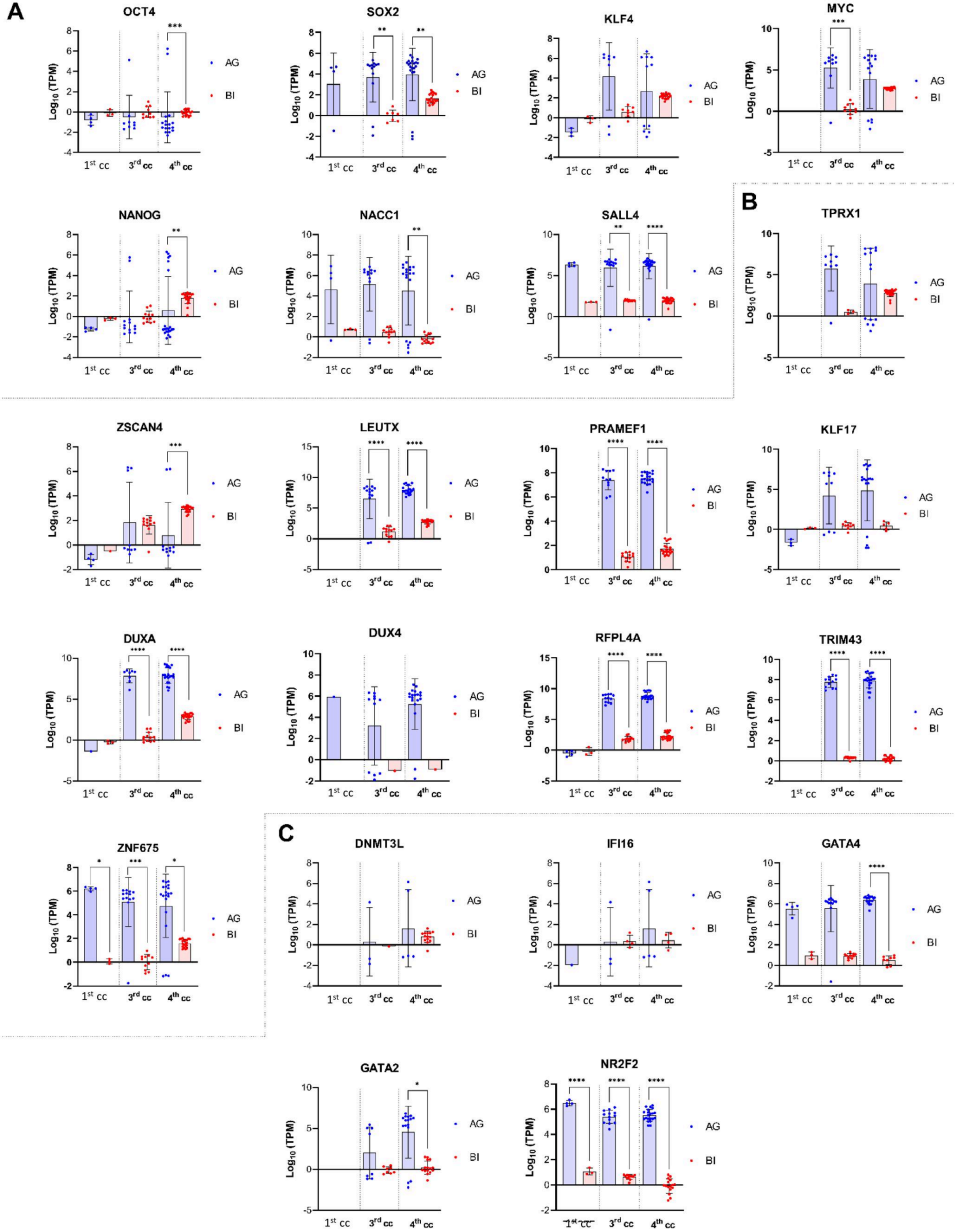
**Differential key gene expression between Androgenotes and Biparental.** The expression of genes related to (**A**) pluripotency, (**B**) zygote genome activation, and (**C**) developmental progression of the embryo (57) was evaluated in individual androcytes (blue dots) from androgenotes (AG) or cells (red dots) from biparental embryos (BI). Gene expression levels are presented on a Log10 scale, as an overall mean ± standard deviation. *P < 0.05. **P < 0.01. ***P < 0.001. ****P < 0.0001. cc, cell cycle.

Within the pluripotency category, the transcription of OCT4, SOX2, KLF4, MYC, NANOG, NACC1, and SALL4 genes was studied throughout the first four cell cycles and compared with biparental embryos obtaining diverse results of which, interestingly, SOX2 and MYC were significantly upregulated in AGs compared to biparentals (Fig. 4A). The genes selected for the ZGA category were TPRX1, ZSCAN4, LEUTX, PRAMEF1, KLF17, DUXA, DUX4, RFPL4A, TRIM43, and ZNF675. The expression of these genes was also compared with biparental embryos (Fig. 4B). The significative overexpression of *LEUTX*, *PRAMEF1*, *DUXA*, *RFPL4A*, *TRIM43*, and *ZNF675* in AG, compared to biparental embryos, reflects the crucial paternal contribution to ZGA. Finally, in the developmental progression category, the genes selected were DNMT3L, IFI16, GATA4, GATA2, and NR2F2, as in the other 2 categories, the transcription results of the 3 cell cycles studied were compared with biparental embryos obtaining different results (Fig. 4C).

## Discussion

We studied human uniparental bioconstructs with exclusive paternal contribution, to characterize their transcriptomic landscape during the first steps of early human development. This study innovatively provides the first longitudinal record of single cell NGS-based transcriptomic profiles in human AG, up to the last permitted stage of *in vitro* culture. By comparing profiles of one- to eight-cell stage androcytes with those blastomeres of correctly fertilized biparental embryos, we began to unveil the paternal contributions to early embryo development.

### Longitudinal transcriptomic profile of human AG

Our data suggest a progressive paternal contribution from the first to the fourth cell cycle reflected in the upregulated gene count and enriched biological processes. These findings align with the ‘immediate EGA’ phenomena described in mice and humans, where reprogramming and initiation of transcription occur shortly after fertilization at the one-cell stage (Abe *et al.*, 2018; Asami et al., 2020). In human preimplantation embryos, fertilization triggers maternal transcription via canonical promoters that regulate splicing of protein-coding transcripts (Perry *et al.*, 2023), ultimately regulating ZGA and EGA (Asami *et al.*, 2022) that is jointly regulated by the maternal and paternal genomes, as recent transcriptomic analysis of human AG and PG evidenced (Leng *et al.*, 2019; Xu *et al.*, 2021; de Castro *et al.*, 2023).

Transcription is positively regulated in one-to-eight-cell stage biparental embryos and PG, but absent in AG (Leng *et al.*, 2019; de Castro *et al.*, 2023). Furthermore, we observed that biparental embryos, exhibited upregulated genes related to mRNA processing and splicing, along with translational initiation, indicating a maternally driven preparation of RNA for the subsequent EGA. We recently showed that AG undergo a primitive genome activation during the one-to-four-cell stage, which is related to initial zygote activation and cell divisions mechanics (de Castro *et al.*, 2023). If human ZGA is activated as early as the one-cell stage in AG but delayed to the eight-cell stage in PG, human ZGA may primarily be initiated by the paternal genome (Yuan *et al.*, 2023). While technical differences in PG and AG bioconstructs generation may partially explain this delay (Yuan *et al.*, 2023), the initial transcriptional peak we detected in AG corroborate the functional male contributions to the EGA.

### Molecular functions upregulated in AG

We detected a significant overexpression of molecular functions such as DNA-binding transcription activation and RNA polymerase II transcription regulatory region sequence-specific DNA-binding in one-to-four cell stage AG. These findings support that the paternal genome acts as an enhancer and promoter of transcription, by transmitting the necessary signals to the RNA polymerase II transcriptional machinery and pre-configuring chromatin for genome activation (Kadonaga, 2004; Liu *et al.*, 2020). Our data also revealed that ZGA markers (*NFYA*, *LEUTX*, *ZSCANs*, *ZNFs*, *MYC*, *TPRX1*, *GATAs*, and *KLFs*) are highly overexpressed in the one-to-four cell stage AG. *NFYA* is a trimeric transcription factor (TF) known for its highly selective binding to the conserved CCAAT element, playing a broader role in controlling mRNA production (Ronzio *et al.*, 2020). The *LEUTX* encodes an enhancer binding protein that acts as a potent transcriptional activator during preimplantation development (Gawriyski, *et al.*, 2023). The *ZSCAN*-family of TFs, particularly *ZSCAN4*, preserves genome and chromosome integrity in preimplantation embryos by promoting DNA repair and chromosome stability (Ko, 2016). Zinc finger cluster genes (i.e., *ZNF280A*, *ZNF479*, *ZNF596*, *ZNF679*, *ZNF705A/B/D/G*, and *ZNF735)* are key regulators of transcription, preimplantation embryo quality and competence (Wang *et al.*, 2023). *MYC* is a universal amplifier of transcription that is particularly linked to the onset of EGA (Perry *et al.*, 2023). *TPRX1*, a TF activated shortly after fertilization, is a critical regulator of early ZGA and embryonic development in humans (Zou *et al.*, 2022). *GATA1/2/6* induce developmental reprogramming and define cell lineage-specifying (Shu *et al.*, 2015). Finally, KLF cluster genes, *KLF10/17/18*, are essential for early human development (Leng *et al.*, 2019) due to their key roles in stem-cell maintenance, cell proliferation, embryonic development, tissue differentiation, and metabolism (Presnell *et al.*, 2015).

### Biological processes upregulated in AG

The nascent transcriptomic peak in AG progressively upregulated and activated genes involved in key embryonic processes (i.e. RNA metabolic and biosynthetic processes, cell differentiation, and development regulation) during the fourth cell cycle. Together, these chemical reactions and pathways produce nucleobases, nucleosides, nucleotides, and nucleic acids for EGA. This hypothesis was supported by the juxtaposition of nucleic acid-templated transcription gene overexpression and gradual downregulation of apoptotic processes beyond the first cell cycle. These findings are consistent with studies conducted in mice (Asami *et al.*, 2023), eight-cell stage human AG (Li *et al.*, 2022; Yuan *et al.*, 2023), and human biparental embryos (Perry *et al.*, 2023). Overall, the AG transcription seems to be fully activated from the onset of mitosis and maintained to the eight-cell stage.

The upregulation of *SOX2* and 26 genes of the PRAME superfamily (*PRAMEF1/2,4-20,25-28P*, *33-36P*) during the early stages of human development is a significant finding. To our knowledge, this is the first report elucidating the differential paternal expression of these crucial genes. *SOX2* is a well-established pluripotency regulator that collaborates with other TFs to regulate stem-cell renewal, differentiation and reprogramming in animal models (Avilion *et al.*, 2003; Tremble *et al.*, 2021) and humans (Novak *et al.*, 2020). In turn, the PRAME family proteins are involved in germline development, by maintaining embryonic stem-cell pluripotency and facilitating primordial germ cell development (Kern *et al.*, 2021). Interestingly, PRAME family members are upregulated in four-to-eight cell biparental embryos (Madissoon *et al.*, 2014) and are functionally significant for normal embryonic development, as their absence leads to early embryonic lethality.

The TFs *DUX4* and *DUXA* hold particularly significance in the context of early embryonic development. Both factors are among the earliest expressed TFs during ZGA (De laco et al., 2017; Hendrickson *et al.*, 2017) and showed progressive overexpression in the third and fourth cell cycle AG. *DUX* family genes mediate the totipotency-pluripotency transition by modifying histones to allow access to pluripotency-associated chromatin sites (Bentsen *et al.*, 2020), and a recent study suggested this positive feedback loop may be required to facilitate embryo cleavage (Ren *et al.*, 2022). Conversely, Bentsen *et al.* (2020) proposed a kinetics-driven model in which *DUX* initiates ZGA and regulates its own termination through a temporally delayed negative feedback loop. Nevertheless, the *DUX* genes are exclusive to eutherian mammals and contribute to placental invasion and cytotrophoblast function by inducing transcription in cleavage-stage embryos (Bosnakovski *et al.*, 2021). Taken together, these findings emphasize the paternal contribution via activation of fundamental TFs.

### Paternal contributions to early embryo development

Transcriptomic comparison of AG and biparental embryos highlighted the significant paternal contribution in subsequent developmental stages, particularly in pluripotency regulation, ZGA, and lineage establishment. The core pluripotency TFs, including *OCT4*, *SOX2*, *KLF4*, *MYC* (Li and Belmonte, 2018) along with translational regulation of *NANOG* (Allègre *et al.*, 2022) exert a vital function in maintaining pluripotency.

Nuclear *SOX2* is expressed during the two-cell stage in mice (Pan and Schultz, 2011) and exhibits a progressive overexpression between the morula and blastocyst stages in humans, contributing to pluripotency and cell differentiation (De Los Angeles *et al.*, 2012; Hagey *et al.*, 2022). The interplay of the *SOX2/OCT4* system is responsible for the first and second cell lineage decisions during embryogenesis and regulates pluripotent stem cells and the early stages of embryogenesis (Rizzino and Wuebben, 2016). Interestingly, *SOX2* is significatively enhanced in AG, compared to biparental embryos, elucidating some level of paternal regulation of pluripotency dynamics. Additionally, the master regulator *MYC* was significantly overexpressed in AG during the third cell cycle, exerting global changes in the basal transcription machinery (Patange *et al.*, 2022). The overactivity of *SOX2* and *MYC* together with *NACC1* (Malleshaiah *et al.*, 2016) and *SALL4* (Tan *et al.*, 2013; Rebuzzini *et al.*, 2021), respectively, associated with stem-cell self-renewal and preimplantation development, highlights the paternal control in early preimplantation human embryos. While *OCT4* and *NANOG* show a minor paternal contribution, previous studies have demonstrated their maternally biased allele expression in PG (Leng *et al.*, 2019), evidencing their maternal effects (Leng *et al.*, 2019).

Our study characterized the longitudinal expression of key ZGA activators in AG, including *TPRX1*, *ZSCAN4*, *LEUTX*, *PRAMEF1*, *KLF17*, *DUXA*, *DUX4*, *RFPL4A*, *TRIM43*, and *ZNF675* (Jukam *et al.*, 2017; Asami et *al*., 2020; Taubenschmid-Stowers *et al.*, 2022; Zou *et al.*, 2022; Yuan *et al.*, 2023). The significative overexpression of *LEUTX*, *PRAMEF1*, *DUXA*, *RFPL4A*, *TRIM43*, and *ZNF675* in AG, compared to biparental embryos, reflects the crucial paternal contribution to ZGA and corroborates findings of *in vitro* ZGA models revealing a transcriptomic signature that encompasses all these factors (Taubenschmid-Stowers *et al.*, 2022). Our data provides evidence of a paternal activation of *LEUTX*, a master regulator (Gawriyski *et al.*, 2023) known for facilitating chromatin modifications, activating key DNA binding enhancers, and inducing expression of developmental TFs, epigenetic modifiers and other markers of the 8-cell stage. We also corroborated the presence of the primate-specific *ZNF675* in eight-cell AG (Yuan *et al.*, 2023) and novelty report the expression at the one-cell stage significatively differs from that of biparental embryos, further substantiating the involvement of *ZNF675* in early embryonic development.

Finally, the comparison of AG and biparental embryo transcriptomic profiles provided insights into lineage establishment. Despite this process being initiated in the fourth cell cycle and crucial for the coordinated development of compaction and cavitation (the two main morphological events during preimplantation stages), the sequence of cell-fate decisions in the preimplantation human embryos remains poorly understood. Data from single-cell transcriptomic analysis have shed light on early differentiation events in human embryos and provided novel markers of developmental progression (Meistermann *et al.*, 2021). Based on the hierarchical scheme of gene expression and lineage signatures, we compared the expression of seven key genes between AG and biparental embryos: *ZSCAN4*, *OCT4*, *DNMT3*, *IFI16*, *GATA2/4*, and *NR2F2*. Remarkably, AG overexpressed genes related to specific lineage establishment, such as *GATA2* for medium/late trophectoderm fate progression, *NR2F2* for polar trophectoderm and *GATA4* for primitive endoderm. In contrast, *ZSCAN4* and *OCT4* were significatively overexpressed in biparental embryos. These preliminary observations suggest the paternal implication in the dynamics of lineage specification. However, it is important to note that some of these genes were found to be overexpressed in PG in our previous paper when we compared with AG (de Castro *et al.*, 2023), so additional research is required to fully understand this complex process.

This study has some limitations, including the ethical considerations regarding human AG culture, and restricted number of single-cells analyzed, which may introduce stochastic effects. AG bioconstructs also have compromised developmental capacity and an irregular transcriptional program compared to biparental embryos (Leng *et al.*, 2019). Thus, caution should be exercised when comparing transcriptomic levels between AG and biparental embryos, as the paternal and maternal genomes do not act independently in normal circumstances. Also, in androcytes, ensuring chromosome formula is challenging since there was no prioritization of chromosome status over single-cell transcriptomics information. Additionally, the impact of AG generation protocols on genome activation remains poorly understood. Furthermore, the transcriptomic analysis includes total poly(A)-mRNA analysis. Thus, it is not possible to discriminate between transcripts actively produced from the sperm cell genome and those already present in the ooplasm. Further research is required to validate the transcriptomic findings in biparental embryos.

In conclusion, our study provides new insights into paternal ZGA activators and the temporal patterns of gene expression in early human development, by demonstrating there is a primitive wave of novel transcriptomic activation, driven by the paternal genome, maintained until the fourth cell cycle. Our findings suggest that hundreds of overexpressed paternal genes influence a broad range of embryo developmental processes, including pre-configuring of the transcription machinery, cell proliferation, differentiation and lineage-specification, along with pluripotency acquisition and maintenance. Finally, the comparison of transcriptomic profiles between AG and biparental embryos highlighted an early paternal contribution to key developmental events. Our study enhances understanding of gene expression patterns in human preimplantation embryos and provides a basis to explore the etiology of gamete-related developmental abnormalities in clinical practice.

## Supplementary Material

deae072_Supplementary_Figure_S1

deae072_Supplementary_Figure_S2

deae072_Supplementary_Table_S1

deae072_Supplementary_Table_S2

deae072_Supplementary_Table_S3

deae072_Supplementary_Table_S4

deae072_Supplementary_Table_S5

deae072_Supplementary_Table_S6

## Data Availability

The data underlying this article are available in Gene Expression Omnibus (GEO) at https://www.ncbi.nlm.nih.gov/geo/, and can be accessed with GSE216501.
