# Supplementary material for: Longitudinal profiling of human androgenotes through single-cell analysis unveils paternal gene expression dynamics in early embryo development
Source: Hum Reprod. 2024 Apr 15;39(6):1186–96. doi: 10.1093/humrep/deae072 (PMC11145015; doi:10.1093/humrep/deae072)
Supplement: deae072_Supplementary_Figure_S1 [file deae072_supplementary_figure_s1.pdf]

OXFORD
human reproduction
Human Reproduction, 2024, 1–1
https://doi.org/10.1093/humrep/deae072
Supplementary Data

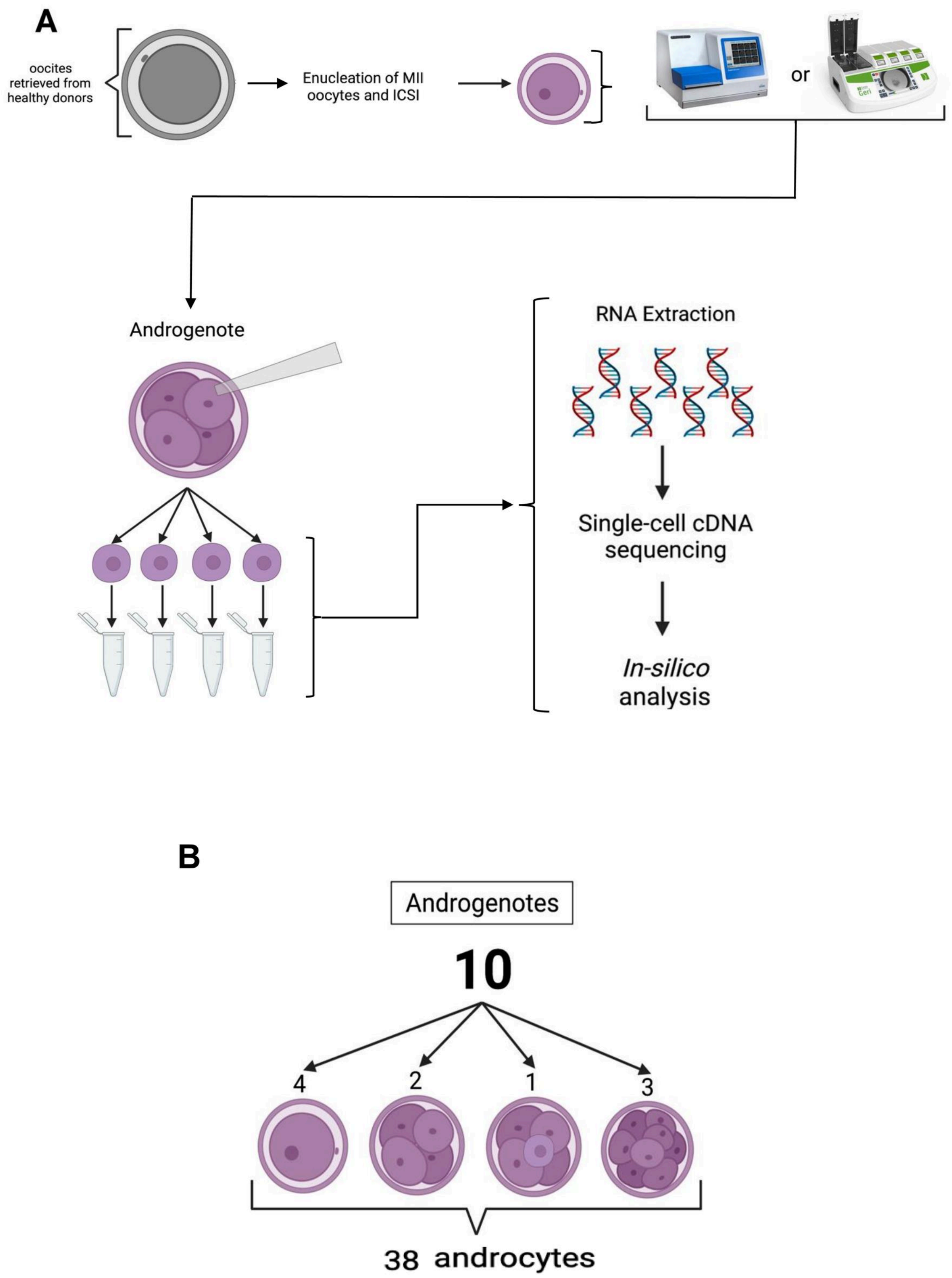

Supplementary Figure S1. Experimental design. (**A**) Healthy and mature metaphase II (MII) donor oocytes (n = 19) were enucleated, subjected to ICSI, and cultured in a time-lapse incubator to generate androgenotes as previously described (ref). (**B**) Of the 10 androgenotes obtained, four were collected at the pronuclear stage (first cell cycle), two were collected at the four-cell stage (third cell cycle), one was collected at the five-cell stage (late third cell cycle), and three were collected at the eight-cell stage (fourth cell cycle). In total, 38 single cells (androcytes) were isolated and analyzed by RNA-sequencing for transcriptomic characterization. Figure created with BioRender.com.
