# Supplementary material for: Longitudinal profiling of human androgenotes through single-cell analysis unveils paternal gene expression dynamics in early embryo development
Source: Hum Reprod. 2024 Apr 15;39(6):1186–96. doi: 10.1093/humrep/deae072 (PMC11145015; doi:10.1093/humrep/deae072)
Supplement: deae072_Supplementary_Figure_S2 [file deae072_supplementary_figure_s2.pdf]

Human Reproduction, 2024, 1–1
https://doi.org/10.1093/humrep/deae072
Supplementary Data

1st vs. 3rd cell cycle

A

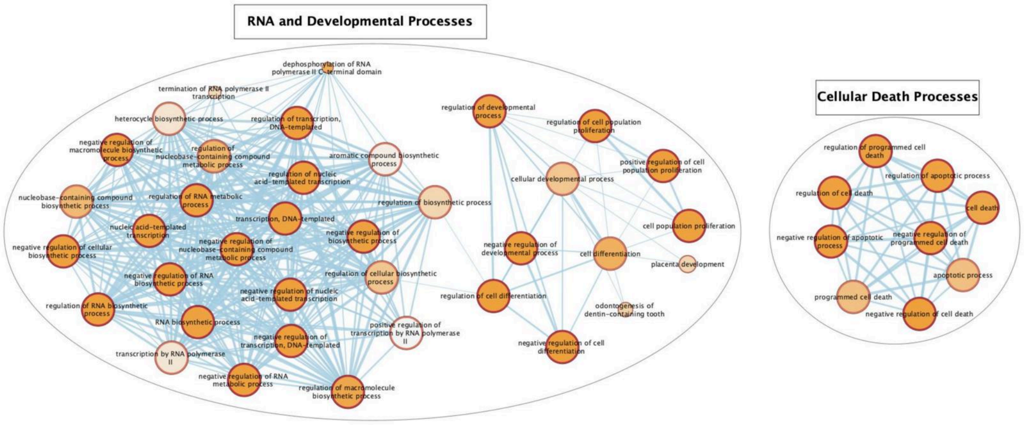

1st vs. 4th cell cycle

B

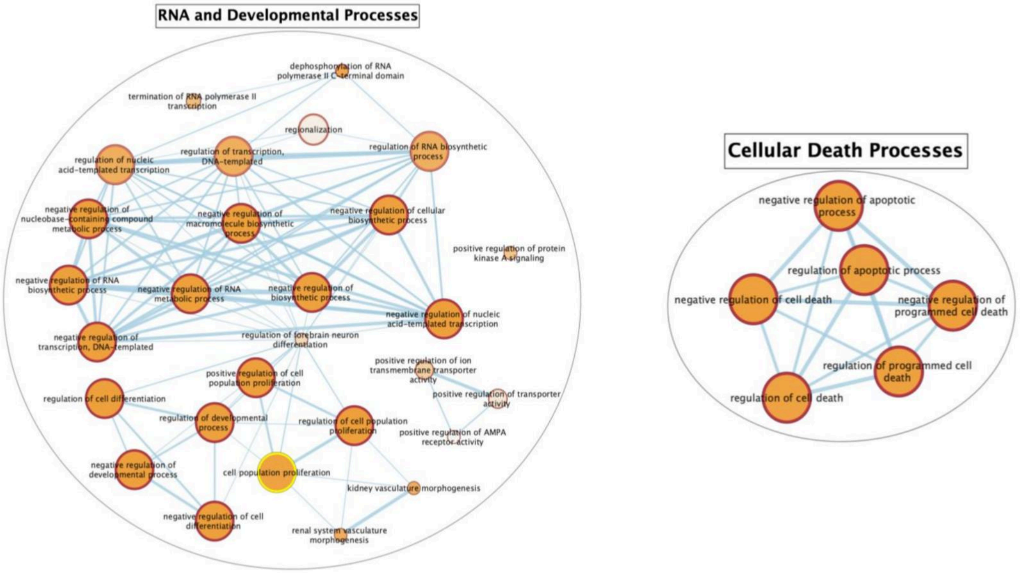

Supplementary Figure S2. Functional enrichment network between Androgenotes. Networks of enriched biological processes in androgenotes at the (**A**) third and (**B**) fourth cell cycles, with respect to the first cell cycle. Orange nodes indicate upregulated gene sets. The intensity of the node color is proportional to the level of differential expression. vs., versus.
